# Comparative efficacy of intraoperative extracorporeal irradiated and alcohol-inactivated autograft reimplantation for the management of osteosarcomas—a multicentre retrospective study

**DOI:** 10.1186/s12957-021-02271-w

**Published:** 2021-05-26

**Authors:** Meitao Xu, Ming Xu, Shuai Zhang, Hanqing Li, A. I. Qiuchi, Xiuchun Yu, Xu Quan Wang

**Affiliations:** 1 Department of Orthopaedics, Southwest Hospital, Third Military Medical University, Chongqing, China; 2 Department of Orthopaedics, The 960th Hospital of Chinese People’s Liberation Army Joint Logistic Support Force, Jinan, China; 3 Department of Orthopaedics, Gui Qian International General Hospital, Guiyang, China

**Keywords:** Alcohol-inactivated, Bone defects, Cobalt-irritated, Osteosarcoma, Reimplantation

## Abstract

**Background:**

Biologic bone reconstruction in limb salvage surgery for the treatment of malignant bone tumours has always been controversial. The various inactivation methods, their convenience and stability, the curative effects elicited and associated costs all need to be considered. This study aimed to compare the clinical efficacy of intraoperative extracorporeal irradiated reimplantation with alcohol-inactivated autograft reimplantation for limb salvage surgery in patients with osteosarcoma.

**Methods:**

We retrospectively analysed 28 patients with osteosarcoma, 14 patients treated with intraoperative cobalt 60 irradiation and reimplantation (group A), and 14 patients treated by alcohol-inactivated autograft reimplantation (group B). The postoperative complications and clinical efficacy of each treatment method were compared by statistical analysis.

**Results:**

The local recurrence rate was 14.3% in each group. Complete bony union was achieved in 64.3% of patients in group A and 71.4% of patients in group B. The overall 5-year survival rate was 71.4% in group A and 78.6% in group B. The mean Musculoskeletal Tumor Society (MSTS) score was 25.33 ± 4.72 (range 15–30) in group A and 24.00 ± 5.85 (range 15–30) in group B, and the mean International Society of Limb Salvage (ISOLS) score was 25.79 ± 5.13 (range 20–36) in group A and 26.14 ± 5.33 (range 20–30) in group B. P < 0.05 was considered to indicate a significant difference. The results showed that the long-term clinical efficacy did not differ significantly between the two methods.

**Conclusions:**

In limb salvage surgery for osteosarcoma, intraoperative extracorporeal irradiation and alcohol-inactivated autograft reimplantation yielded equivalent outcomes. The alcohol-inactivated method may be a much more convenient and inexpensive way to reconstruct bone defects. Additional studies as well as more case studies are needed to fully evaluate the clinical efficacy and safety of this treatment method.

## Background

Osteosarcoma is the most common primary malignant bone tumour in adolescents [1], for which a combination of surgical treatment and neoadjuvant chemotherapy has been administered over the past few decades [2]. Many clinical trials are being conducted to treat osteosarcoma using a variety of strategies, such as osteoarticular or intercalary allografts, allograft-prosthetic composites, custom-made or modular prostheses, distraction osteogenesis and arthrodesis with autogenous or allogenic bone [3–6], with comprehensive management including limb salvage surgery and systemic chemotherapy.

Currently, 90 to 95% of patients with malignant bone tumours can be safely treated with limb salvage surgery. The postoperative recurrence rate is low, and the survival rate is equivalent to that of amputation. However, the reconstruction of bone defects after resection is challenging [4].

By conducting a literature review and summarizing our relevant clinical experience with biologic bone reconstruction in the treatment of malignant bone tumours in the limbs, we conducted a retrospective case-control study to compare the clinical efficacy of intraoperative extracorporeal irradiated and alcohol-inactivated treatment for the management of osteosarcoma.

## Methods

The study was approved by the Institutional Ethics Review Board of the First Affiliated Hospital at Third Military Medical University (KY201779) and the Ethics Committee of the 960th Hospital of Chinese People’s Liberation Army Joint Logistic Support Force. Written informed consent was obtained from all patients or their guardians and retrospectively registered. Intraoperative cobalt 60 extracorporeal irradiated autograft reimplantation (group A) was performed at the First Affiliated Hospital at Third Military Medical University, and alcohol-inactivated autograft reimplantation (group B) was performed at the 960th Hospital of Chinese People’s Liberation Army Joint Logistic Support Force.

We retrospectively reviewed the charts of 28 patients with osteosarcoma of the limbs who were treated between February 2004 and October 2012. The biological reconstruction method used for the bone defects in limb salvage surgery was identified from the medical records, and the average age of the patients was 16.36 ± 7.03 years (range 7–38 years). The radiography and magnetic resonance imaging (MRI) scans of the affected limb were reviewed. Pulmonary computed tomography and Tc-99 bone scintigraphy (ECT) scans were performed to determine whether metastasis was present. Pathological diagnoses were confirmed by needle biopsy at the initial visit. All 28 patients underwent neoadjuvant chemotherapy, including two to four sessions of preoperative chemotherapy with the cisplatin, ifosfamide and adriamycin (DIA) chemotherapy regimen [5]. This programme usually took 6–8 weeks to complete. The tumour boundary was determined by MRI, ECT, and X-ray before and after chemotherapy.

The inclusion criteria were as follows: from February 2004 to October 2012, osteosarcoma of the limb was treated with cobalt 60 irradiation and reimplantation or with alcohol-inactivated autograft reimplantation. Osteogenesis, sclerosis or small bone dissolution was detected by imaging. The resection boundaries of the tumour were stage IIb according to the Enneking staging system [6], and the neoplasm did not invade the articular surface, with at least 2 cm of healthy bone remaining for proper internal fixation or bone cement filling. The patient and his or her family members voluntarily agreed to the surgical plan. The exclusion criteria were as follows: the neoplasm mainly invaded the articular surface; distant metastases were present; the criteria for extensive resection or boundary resection were not met; and the patient and his or her family chose tumour prosthesis reconstruction, allograft reimplantation or amputation surgery. All 28 patients with osteosarcoma had stage IIb tumours according to the Enneking staging system [6].

**Fig. 1:**
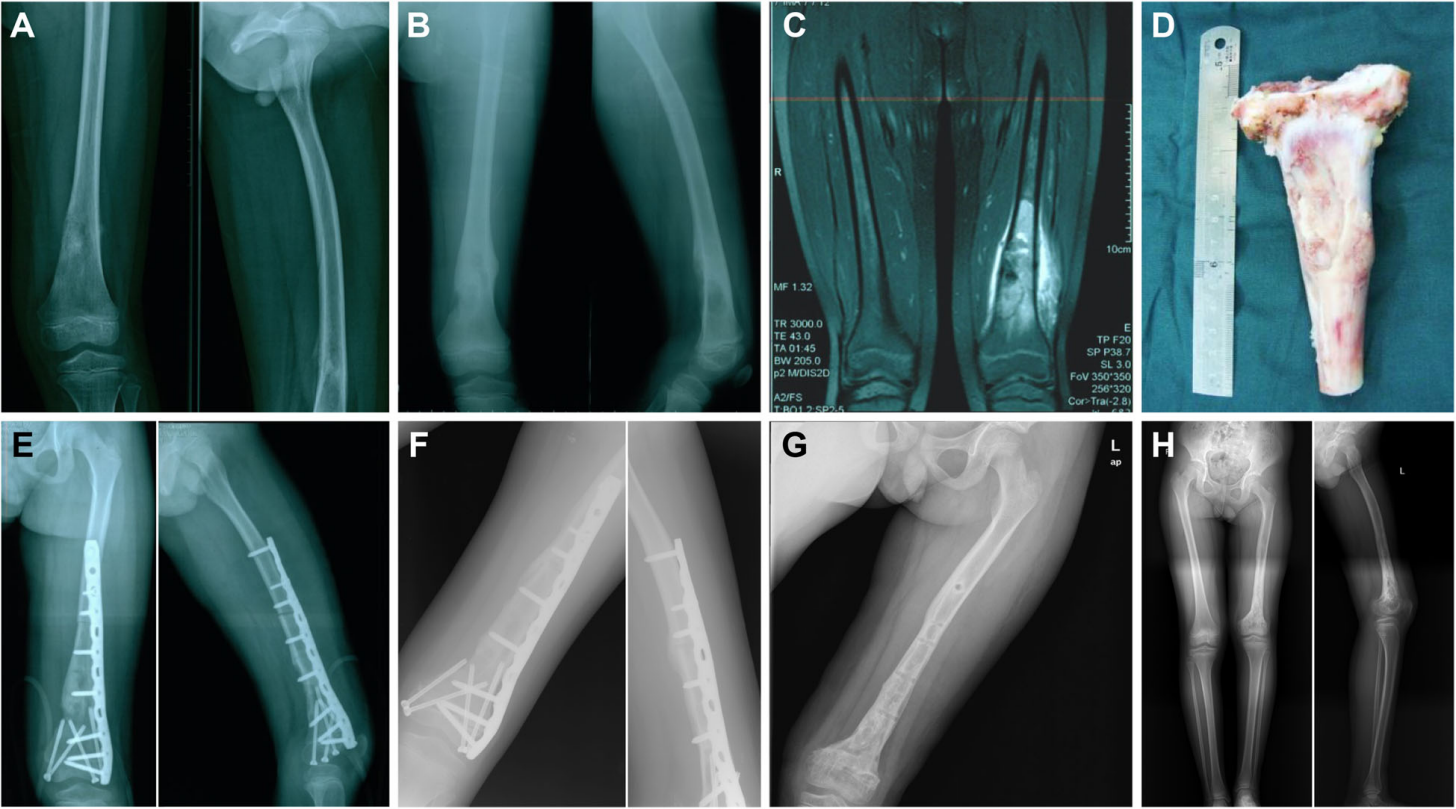
**A** Prechemotherapy X-ray examination showing osteolytic bone destruction in the left distal femur of a 7-year-old male patient who underwent joint preservation intraoperative extracorporeal inactivated autograft replantation for osteosarcoma of the distal femur. Bone destruction was observed on the medial side, with a local soft tissue mass and periosteal reaction. **B** Two months after chemotherapy, the X-ray examination revealed that osteolytic bone destruction had markedly decreased in the distal femur. The soft tissue mass had disappeared. **C** Postchemotherapy coronal MRI scan shows a mix of high and low signals inside the medullary cavity. The surrounding soft tissue and bone marrow response area were clearly demarcated. **D** After 30 min and 50 Gy dose irradiation by cobalt 60, the 13 cm length of inactivated autogenous segment was returned for reconstruction. **E** The radiographs taken 1 week after surgery showed apparent reduction of the distal femur by solid internal fixation and joint preservation. **F** Four months after surgery, the X-ray examination revealed bone healing and a normal joint space. **G** The X-ray examination showed excellent bone healing, and the internal fixation device was removed 2 years after limb salvage surgery. **H** At the 4-year follow-up, the full-length X-ray of the left lower extremity showed that the extremity was approximately 5 cm shorter than normal

For the intraoperative cobalt 60 extracorporeal irradiated autograft reimplantation group (group A), the soft tissues of the autogenous tumour segment were thoroughly removed, and the bone segment was aseptically packed and received cobalt 60 irradiation immediately (group A). After 50 Gy dose irradiation for 30 min, the inactivated autogenous bone segment was returned immediately for reconstruction surgery. The medullary bone tissue of the extracorporeal irradiated autograft was scraped off and sent for pathological confirmation of negative findings. Multiple samples were collected from the surgical area and sent for frozen pathological examinations to determine whether there was residual tumour at the cutting edge to ensure the safety of the resection boundaries at least marginally (or extensively). Moreover, bleeding in the surgical wound was completely stopped, and the wound was immersed and rinsed repeatedly with normal saline to keep the area clean and sterile. Finally, the inactivated segment was sent back to the operating room for reimplantation by internal fixation (Fig. 1A–H). Bone cement was used to fill any significant bone lacunar defects if necessary.

**Fig. 2:**
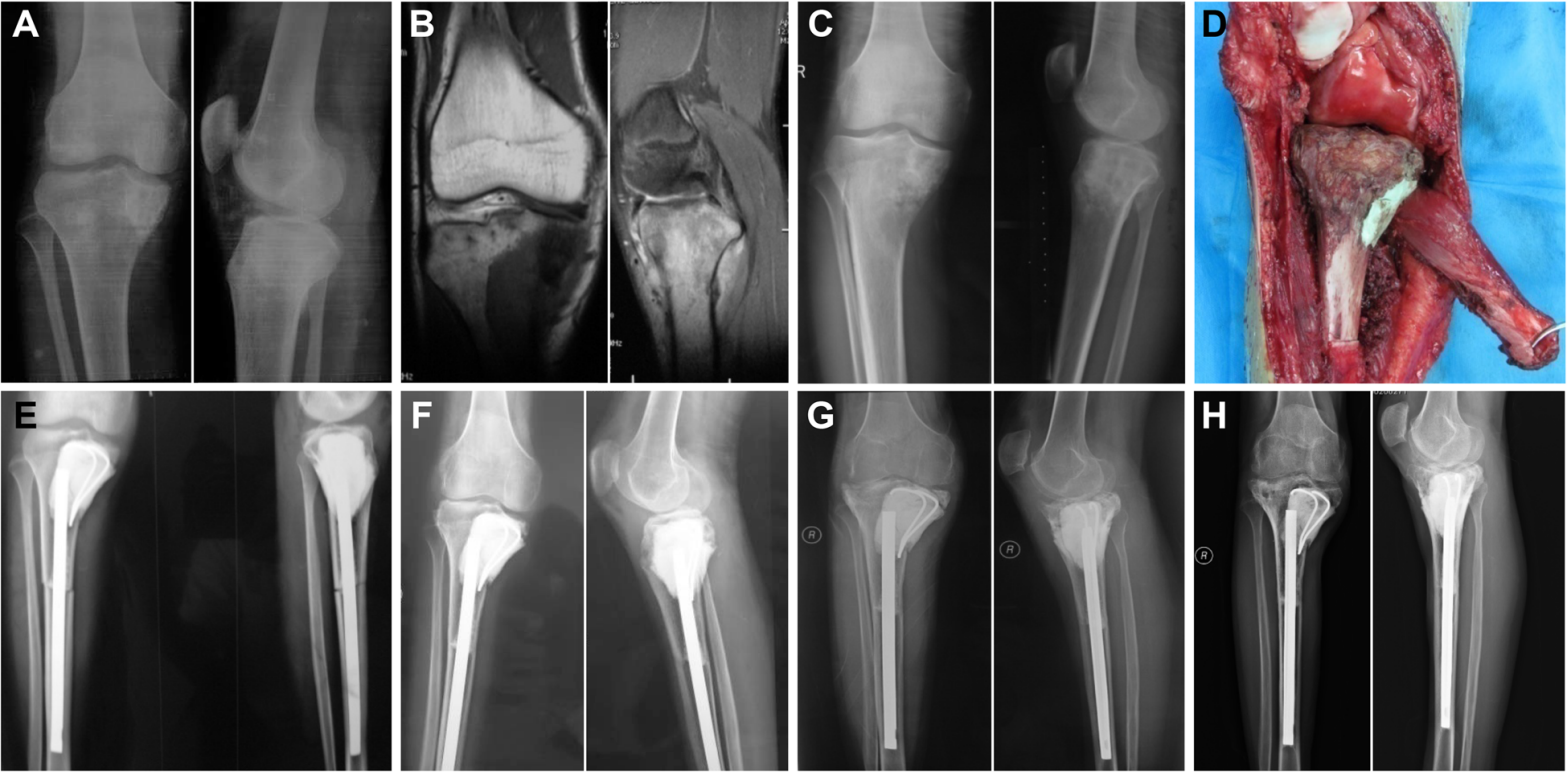
**A** Prechemotherapy X-ray examination showing osteolytic bone destruction in the right proximal tibia of an 18-year-old male patient who underwent joint preservation alcohol-inactivated autograft replantation for osteosarcoma. **B** Prechemotherapy coronal and sagittal MRI scans show a mix of high and low signals inside the medullary cavity of the tibia and surrounding soft tissue. **C** Two months after chemotherapy, the X-ray examination revealed that osteolytic bone destruction had markedly decreased. **D** The photo taken during surgery showed that the alcohol-inactivated autograft segment was filled with bone cement in the bone defect of the medial tibial plateau, replanted for reconstruction and firmly fixed by intramedullary nail fixation. **E** Radiographs taken 1 week after surgery showed solid internal fixation and joint preservation of the knee. **F** At 1 year after surgery, the X-ray examination revealed bone healing, but articular space narrowing was observed. **G** X-ray taken 3 years after limb salvage surgery. **H** At the 7-year follow-up, the X-ray examination of the right knee showed the condition of the alcohol-inactivated autograft segment. The subchondral bone was partially resorbed and fractured, narrowing of the space was observed, and arthrosis was indicated

For the alcohol-inactivated autograft reimplantation group (group B), the tumour was surgically resected according to the standard guidelines for resection of malignant tumour segments. Soft tissue and the extraosseous tumour were removed, and the medullary cavity of the autograft bone tumour segment was drilled through. Pathological diagnostic testing was performed following the same procedure as that for group A. Screw holes for the intended fixation method were preliminarily created. The tumour segment was inactivated by soaking it with 99% alcohol for 30 min, and it was retrieved and flushed with 3000 ml of physiological saline. Bone cement was pressurized into the inactivated bone segment marrow cavity or bone defect. Excess or leaking bone cement was removed, and then the autograft segment was reimplanted into the limb defect with solid and effective internal fixation. Screws were inserted into the previously prepared screw holes, and intramedullary screws or steel plates were selected for internal fixation (Fig. 2A–H).

During and after the operation, pathological testing confirmed that there was no residual tumour at the edge of the segment or live tumour cells in the inactivated bone. Routine prophylactic antibiotics were administered for 48 h, and incision indwelling drainage was performed postoperatively. A brace was used during rehabilitation for protection or support if necessary. Bed rest with non-weight-bearing activity was prescribed for 4 to 6 weeks, after which the patient was allowed to carry out partial weight-bearing activities using two crutches; the patient was gradually allowed to perform full weight-bearing activities within 3 to 6 months after the operation. All patients received nutritional support and were told to avoid strenuous exercise.

All the patients were followed up 1, 3, 6, 9 and 12 months after surgery and then once a year thereafter. The end of the follow-up period was determined by patient death or loss to follow-up. None of the 28 patients were lost to follow-up. Clinical complications and limb function were observed. Radiology (X-ray and CT) was used to evaluate the extent of distant metastasis, local recurrence, bone healing, limb function, and internal fixation. Limb function was evaluated according to the Musculoskeletal Tumor Society (MSTS) functional scoring system. The conditions of the inactivated bones were assessed according to the International Society of Limb Salvage (ISOLS) image scoring criteria [7].

All analyses were performed using the SPSS statistical software package (version 14.0, SPSS Inc., Chicago, IL, USA). Continuous variables were analysed by the t test, and categorical variables were compared using the chi-square test. The MSTS and ISOLS scores were compared between the two groups. *P* < 0.05 was considered to indicate a significant difference. Complications were characterized by descriptive statistics according to the current version of the ISOLS classification system [8]. The overall survival rate was determined and compared using the Kaplan-Meier method.

## Results

Between February 2004 and October 2012, 28 patients were included in this research, including 14 males and 14 females with an average age of 16.36 ± 7.03 years (range 7–38 years). Eleven neoplasms were located in the distal femur, 11 were located in the proximal tibia, and six were located at other sites (four in the distal tibia, one in the proximal humerus and one in the proximal radius). All patients were followed up for 98.07 ± 55.29 months (range 6–182 months). In group A, the mean follow-up time at the last visit was 133.43 ± 20.55 months (range 6–182) months. In group B, the mean follow-up time at the last visit was 122.50 ± 14.06 months (range 16–150). In total, 25 patients underwent wide resection, and three patients underwent marginal resection. The average length of the extracorporeal irradiated autograft was 17.64 ± 4.77 cm (range 12–29), and that of the alcohol-inactivated autograft was 16.00 ± 4.08 cm (range 12–24 cm) (Table 1).

**Table 1:** Demographic characteristics of the patients

Group	Group A (*n* = 14) Extracorporeal irradiation treatment	Group B (*n* = 14) Alcohol-inactivated treatment	*P* value
Sex			
Male	6	8	0.706^a^
Female	8	6	
Age, years (mean)	17.14 ± 8.87	15.57 ± 4.78	0.564^b^
Tumour location			
Distal femur	5	6	0.291^c^
Proximal tibia	4	7	
Other	5	1	
Autograft length (mean, cm)	17.64 ± 4.77	16.00 ± 4.08	0.336^d^
Follow-up (mean, months)	133.43 ± 20.55	122.50 ± 14.09	0.590^e^

^a^There was no difference in sex between the two groups (according to Fisher’s exact test, *P* > 0.05)

^b^There was no difference in tumour location between the two groups (according to the independent-samples t test, *P* > 0.05)

^c^There was no difference in age between the two groups ( according to Fisher’s exact test, *P* > 0.05)

^d^There was no difference in autograft length between the two groups (according to the independent-samples t test, *P* > 0.05)

^e^There was no difference in the follow-up duration between the two groups (according to the Kaplan-Meier test, *P* > 0.05)

Postoperative complications of the autografts included soft tissue failure in 7.1% (1/14) of patients, aseptic loosening in 28.6% (4/14), structural failure in 14.3% (2/14) and infection in 14.3% (2/14) in each group. Complete bony union was achieved in 64.3% (9/14) of the patients in group A and in 71.4% (10/14) of the patients in group B. The bones were mostly healed within 8 to 14 months after surgery. The limb salvage rate was 78.6% (11/14) in group A. One patient with right proximal tibia osteosarcoma suffered from a severe purulent infection 2 months after surgery and underwent treatment with surgical debridement followed by sensitive antibiotics for 6 weeks, but the curative effect was poor, and amputation surgery was carried out. One cases of local recurrence due to the adjacent soft tissue not originating from the transplanted autograft was observed, and amputation was carried out at the patient’s insistence at 5 months after surgery. In another patient with osteosarcoma of the left distal tibia, local recurrence occurred in the soft tissue of the ankle at 62 months after surgery. As a result, lower leg amputation was carried out. The limb salvage rate was 85.8% (12/14) in group B. One patient with left distal femur osteosarcoma underwent amputation because of locally recurrent soft tissue tumours, and the graft was invaded 29 months after surgery. Another patient with left distal femur osteosarcoma with a persistent postoperative infection, a poor MSTS score and structural failure at 36 months after surgery underwent subsequent lower thigh amputation. Regarding tumour progression, none of the patients exhibited bony progression. Two cases of soft tissue local recurrence were observed in each group, with a local recurrence rate of 14.3% (2/14).

At the end of the follow-up period, the mean MSTS score was 25.33 ± 4.72 (range 15–30) in group A and 24.00 ± 5.85 (range 15–30) in group B, and the mean ISOLS score was 25.79 ± 5.13 (range 20–36) in group A and 26.14 ± 5.33 (range 20–30) in group B. There was no significant difference in the incidence of tumour recurrence or long-term clinical efficacy between the extracorporeal irradiation and alcohol-inactivated methods for biological reconstruction in limb salvage surgery for osteosarcoma (Table 2).

**Table 2:** Comparison of the MSTS scores and ISOLS scores between the two groups at the last follow-up

Group	Group A (*n* = 14) Extracorporeal irradiation treatment	Group B (*n* = 14) Alcohol-inactivated treatment	*P* value
^※^MSTS score	25.33 ± 4.72	24.00 ± 5.85	0.539^a^
^※^ISOLS score	25.79 ± 5.13	26.14 ± 5.33	0.858^b^

^a^There was no difference in the MSTS score between the two groups (according to the independent-samples t test, *P* > 0.05)

^b^There was no difference in the ISOLS score between the three groups (according to the independent-samples t test, *P* > 0.05)

^**※**^Musculoskeletal Tumour Society (MSTS) score, International Society of Limb Salvage (ISOLS) score

**Fig. 3:**
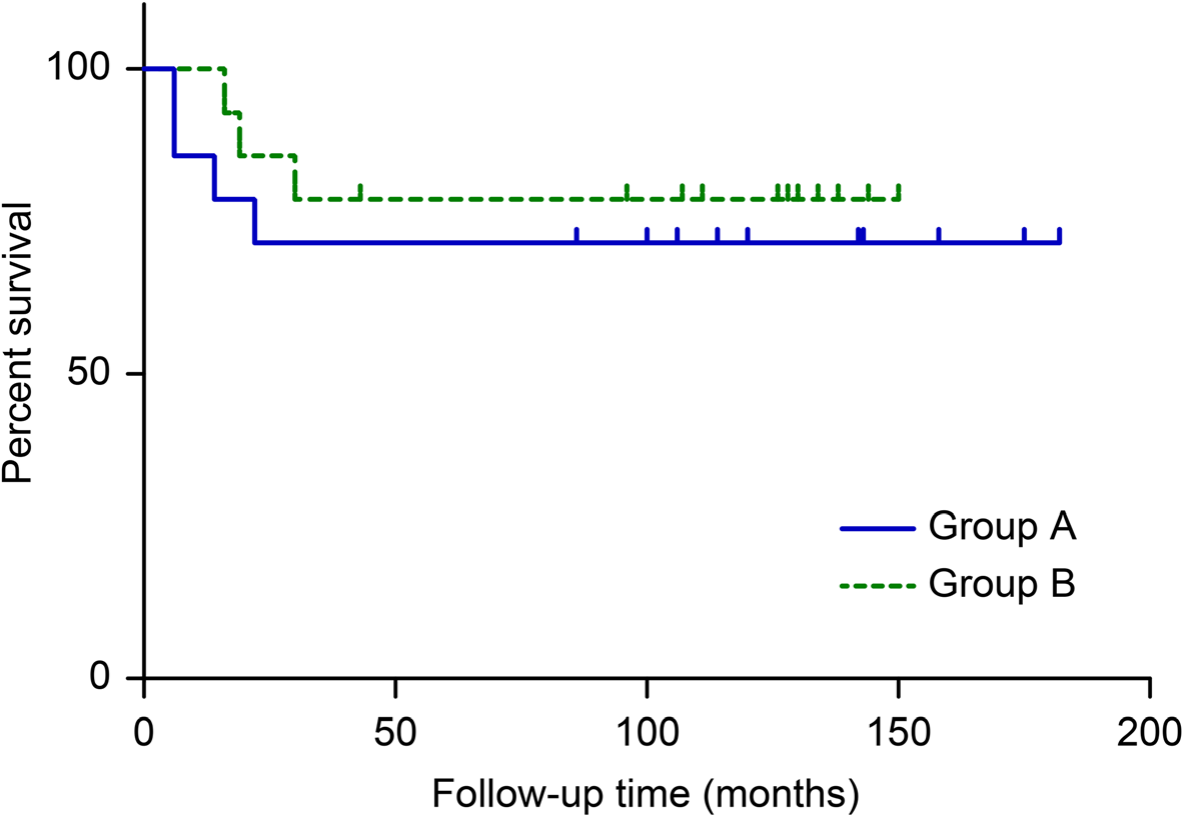
The Kaplan-Meier modelling results for 5-year overall survival are shown

The overall mortality rate was 25.0% (7/28). Seven patients died of pulmonary metastases, with a metastasis rate of 28.6% (4/14) in group A and 21.4% (3/14) in group B, and ten patients survived for more than 5 years, with an overall 5-year survival rate of 71.4% (10/14) in group A and 78.6% (11/14) in group B. The 5-year survival rates of the two groups were calculated according to the Kaplan-Meier survival curve, and no difference in survival was found between the extracorporeal irradiation and alcohol-inactivated groups (*P* > 0.05) (Fig. 3).

## Discussion

Osteosarcoma is the most common highly malignant bone tumour in adolescent patients and has high mortality and disability rates. As neoadjuvant chemotherapy and imaging techniques continue to advance and reconstruction methods continue to be developed, limb salvage is becoming the preferred treatment. Immediate recycling of the resected bone segment in biological limb salvage reconstruction is an option after wide resection [9]. Although some osteosarcomas amputation techniques have been improved [10], compared with amputation, limb salvage can yield similar 5-year survival and disease-free survival rates, and it is more psychologically accepted by patients [11]. Due to advances in chemotherapy and surgery, osteosarcoma is no longer considered an almost universally fatal disease, and the majority of patients survive with a meaningful quality of life [12].

Selecting the appropriate treatment programme for bone defect reconstruction is challenging for surgeons. The reconstruction methods for bone defects after resection of malignant bone tumours include the use of manufactured tumour-type artificial joints, biological constructs, bulk allografts, a combination of allograft-prosthetic composites and bone autografts [13, 14]. Although prosthesis replacement is widely used at present, many complications can arise, such as infection, loosening and fracture, especially in young adult patients who have high functional requirements. Furthermore, patients who perform high-intensity activity or have a high level of physical activity experience a higher failure rate. 3D-printed prostheses can also be used but are much more expensive and complicated procedures than those listed previously [15]. In 2019, Zhao et al. [16] conducted a systematic review of 33 studies on tumours affecting the distal tibia published in PubMed and EMBASE databases. Among the 337 cases included, biological reconstruction methods yielded better functional outcomes (78.4% vs. 72.2%, P = 0.017) than nonbiological prosthetic reconstruction methods. There are many problems with the use of allografts; for example, allografts can lead to the spread of infection, can elicit an immune response and can conflict with the patient’s social or religious beliefs, especially in Asian countries [17, 18]. Moreover, high complication rates (70%) and graft rejection rates (60%) have been reported for more than 10 years in patients with limb sarcoma following allograft reconstruction [19]. Furthermore, biological reconstruction might be the optimal method for reconstruction. It has been considered that after resection of the malignant bone tumour, the patient’s own resected tumour segment (after inactivation and reimplantation) could be used as an autograft in bone defect reconstruction. This technique will probably play a future role in the management of osteosarcoma, and the use of allografts could be replaced in some settings [16], especially in countries where foreign bone is difficult to obtain [20]. Reconstruction with pasteurized autograft is a feasible method for treating periacetabular malignant bone tumours, with satisfactory oncological and functional outcomes and a relatively low incidence of complications [21].

Extracorporeal irradiation and freezing are two common techniques used to eliminate residual tumour cells in autografts for limb salvage surgery with the biological reconstruction of bone defects. Acceptable survival rates and satisfactory levels of function have been observed in some studies. In 1968, Spira and Lubin [22] first reported the application of extracorporeal irradiation in limb salvage treatment for malignant bone tumours. Compared with prosthetic reconstruction, this method was found to be more economical, and biological reconstruction was more acceptable to patients and their families [23, 24]. Within the past 50 years, several oncologists have reported this inactivation method, but due to the widespread use of limb salvage surgery for malignant bone tumours, there are still many scholars who prefer and have interest in this technique [13, 25, 26]. A comparative study was performed to determine the effect of intraoperative extracorporeal irradiation and freezing treatments of tumour-bearing autografts, and no differences between the groups were found in the total proportion of patients achieving union at 6, 9, 12 and 18 months [9]. Radiographic evaluations did not show any differences in the average scores of the compared criteria. Tsuchiya et al. [27] used liquid nitrogen to freeze and inactivate autologous tumour bone in 28 cases of malignant bone tumours with an average follow-up time of 28.1 months (range 10–54); bony union was observed at a mean of 6.7 months after the operation in 26 patients (92.8%), and non-union occurred in two patients. Heat treatment can not only reduce bone strength but also lead to a loss of bone induction ability. Jeon et al. [28] treated 15 patients with distal femoral osteosarcoma with high-pressure steam inactivation of autologous bone reimplantation and followed them up for an average of 56 months (35–78 months). After surgery, five patients presented with non-union of bone, and three patients presented with a loose prosthesis; no patients suffered from infection.

The traditional biological bone reconstruction model for limb salvage therapy, extracorporeal irradiation, has been frequently reported, while the alcohol-inactivated technique has rarely been reported. Complications such as graft fracture, infection and non-union have an obviously negative effect on survival of the whole graft. The effectiveness of an alcohol-inactivated bone reimplantation technique for tumour eradication has not yet been elucidated. Thus, based on findings from a previous study, we developed a joint-preserving limb salvage approach for the treatment of osteosarcoma of the distal femur that involved the reimplantation of alcohol-inactivated bone. Alcohol might serve two functions in autografts: to kill microbes as well as tumour cells and to prevent interference in the process of creeping substitution in the host [29]. Ten patients with Enneking stage IIb osteosarcoma were treated by alcohol-inactivated autograft reimplantation with joint preservation. The patients were followed up for a mean of 34 months, and all patients achieved first-stage healing, with a mean MSTS functional score of 23 (77%) [30]. Our preliminary findings indicate that alcohol inactivation is a feasible approach that may help preserve the important structures of the joint and prevent the long-term complications that can occur with endoprosthetic replacement [31]. Additional studies are needed to fully evaluate the efficacy and safety of this surgical approach. By comparing the clinical efficacy of the two methods, our study provides valuable reference information for the clinical treatment of malignant bone tumours. In this study, in the group of 14 osteosarcoma patients treated with alcohol-inactivated autograft reimplantation, the 5-year survival rate was 71.4% (10/14), which is lower than those in the groups of patients treated with extracorporeal irradiation (82.8%) and freezing treatment (84.4%) [13].

Compared with other biological bone reconstruction methods, the alcohol-inactivated method is not only safe and effective at killing tumour cells but also more economical and convenient, yielding the same shape for reimplantation [31]. The disadvantage is that it requires a long period of time to complete revascularization and to achieve normal bone union with the surrounding bone tissue. Moreover, the adjacent joint may exhibit cartilage degeneration and joint relaxation complications. The healing process of autograft bone reimplantation is the result of absorbance, crawling and superseding, which may be the main osteogenic pathways at the bone junction, and the femur healing time is faster than the tibia healing time [32]. In our study, union was most commonly detected within 8–14 months after surgery, which is similar to the findings of a study by Wu et al. [9], who performed a comparative study to observe the effect of intraoperative extracorporeal irradiation and freezing treatments. Ogura et al. [33] conducted a retrospective review of 11 patients undergoing reconstruction using a devitalized autograft, deep freezing and a vascularized fibula graft composite for lower extremity malignant bone tumours. A shorter union time (7 months) was reported for the autografts treated with freezing. One advantage of freezing treatments including extracorporeal irradiation, autoclaving and pasteurization is that bone morphogenetic protein (BMP) is preserved in the inactivated bone [34]. Investigations into whether there are differences in the efficacy of the three methods of autologous bone inactivation (extracorporeal irradiation, freezing or alcohol inactivation) have not been conducted in a controlled clinical study setting. In addition to the influencing factors of different inactivation methods, many other factors, such as the damage or quality of grafts, sites of tumours, reconstruction methods, stabilization during reconstruction, tumour local recurrence and infectious complications, may affect the process of graft osteogenic healing. All these relevant factors need to be considered and incorporated into the design of future studies.

The total complication rate (including tumour progression) for irradiation treatment in this study was 42.9% (6/14). Wu et al. [9], in an early, large-sample-size and controlled study, reported a complication rate of 44% (35/79). In the patients receiving alcohol-inactivated autografts, we found a complication rate of 42.79% (6/14), which was slightly higher than our previously reported rate of 40% (4/10), with one case of local recurrence and three cases of fracture of the inactivated bone or bending or breakage of the intramedullary nail [33]. Infections occurred in 14.3% (2/14) of the patients in each of our groups, which is higher than the rates reported by Wu et al. [9], namely, 8% (6/79) in the irradiation group and 5% (4/85) in the frozen autograft group. The main concern regarding the alcohol inactivation technique is its safety and the potential risk of recurrence, although it has been shown to be safe and effective at killing tumour cells in our preliminary findings for the management of osteosarcoma of the distal femur [30] and alcohol-inactivated autograft-prosthesis composites for grade III giant cell tumour treatment [32]. Even with the clinical application of neoadjuvant chemotherapy and technical improvements in limb salvage surgery, local recurrence occurred in 11.6% of all patients, which was similar to that reported in a study by Bielack et al., in which 12.4% of patients presented with distant metastases of osteosarcoma [35]. The rate of local recurrence was the same between the irradiation group and the alcohol inactivation group in our study, at 14.3% (2/14); in the study by Wu et al., the overall tumour recurrence rate was 15% (12/79) in the group treated with extracorporeal irradiation and 11% (9/85) in the group treated with freezing [9]. No cases of tumour recurrence originating from inactivated bone were observed. This finding proved that either 50 Gy irradiation or 30 min of alcohol inactivation in autografts can yield similar levels of efficacy in eradicating tumour cells. The implantation of tumour cells at the time of surgery is thought to be one of the causes of local recurrence [36]. In terms of survival, no differences between the patients who underwent reconstruction with composites and those who underwent reconstruction with intercalary grafts have been reported [37], consistent with the findings of our study.

Although satisfactory outcomes were obtained in this study, several limitations exist. First, this was a retrospective study with a short follow-up period and small sample size, which may affect the reliability of the results. Additional studies involving larger patient cohorts and longer follow-up periods are needed to fully evaluate the efficacy and safety of this treatment approach. Second, selection bias associated with the surgical methods may have affected the clinical outcomes. Alcohol-inactivated bone requires a longer time to revascularize and integrate with the surrounding bone, so bone healing occurs over a prolonged period after surgery. New combined biological methods should be explored and adopted in the future [38].

## Conclusions

In the patients with osteosarcoma in this study, intraoperative extracorporeal irradiation and alcohol-inactivated autograft reimplantation yielded similar outcomes. For biological reconstruction, compared with the usual choice of traditional irradiation inactivation and freezing using liquid nitrogen therapy, alcohol-inactivated reimplantation could be a more convenient and economical way to reconstruct bone defects for osteosarcoma. Additional studies with more cases and longer follow-up periods are needed to fully evaluate the clinical efficacy and safety of alcohol-inactivated reimplantation.

## Data Availability

All data used in the study are available at the request of the editors and reviewers.
